# Supplementary material for: Causal Relationship between Aging and Anorexia Nervosa: A White-Matter-Microstructure-Mediated Mendelian Randomization Analysis
Source: Biomedicines. 2024 Aug 16;12(8):1874. doi: 10.3390/biomedicines12081874 (PMC11351342; doi:10.3390/biomedicines12081874)
Supplement: Supplementary file 1 [file biomedicines-12-01874-s001.zip › Supplementary Figure/Figure S9/Leaveoneout plot of MR analysis for ubm-b-1513 to AN.pdf]

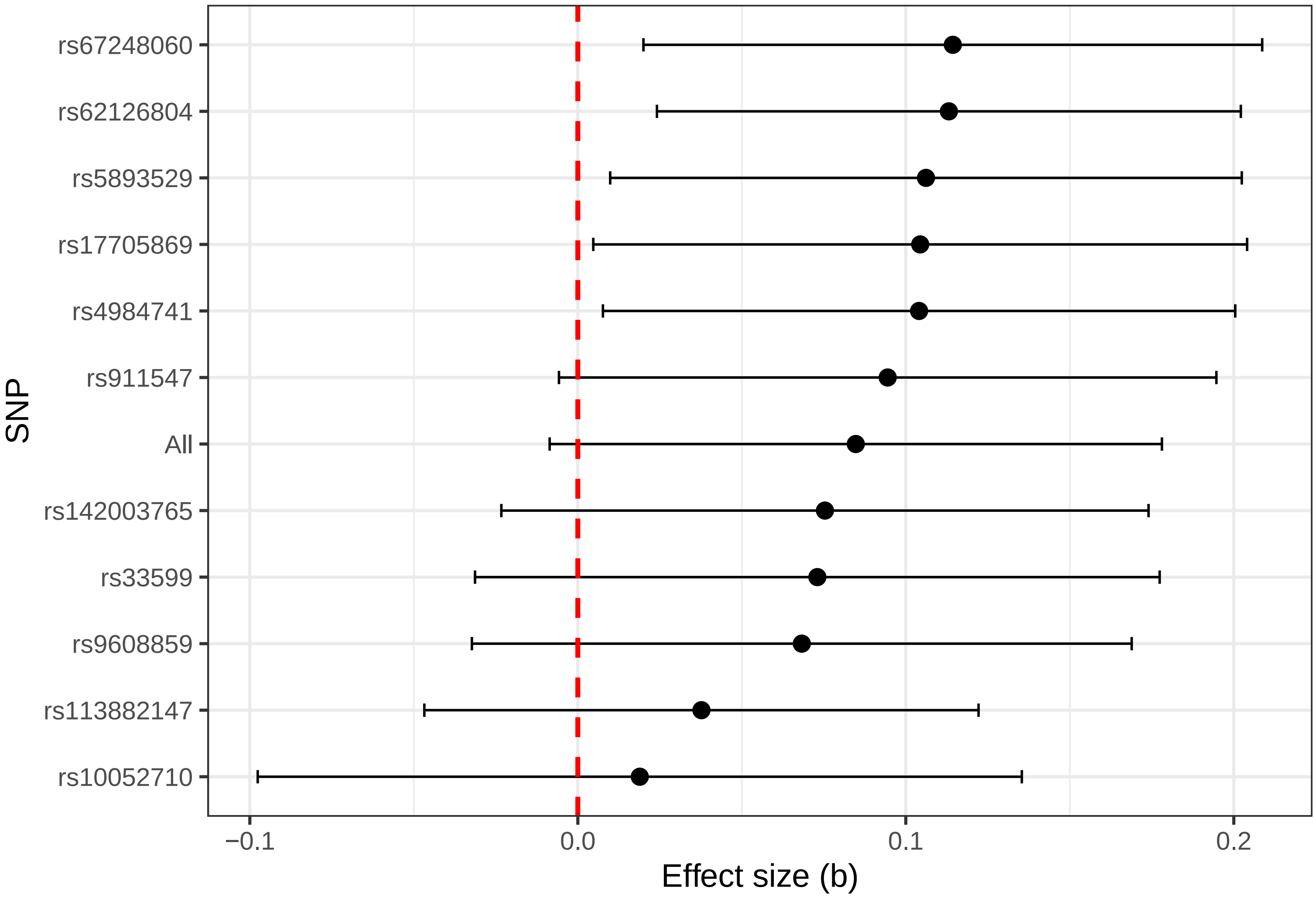

rs67248060
rs62126804
rs5893529
rs17705869
rs4984741
rs911547
All
rs142003765
rs33599
rs9608859
rs113882147
rs10052710
SNP
−0.1
0.0
0.1
0.2
Effect size (b)
